# Supplementary material for: Slicing Spheroids in Microfluidic Devices for Morphological and Immunohistochemical Analysis
Source: Micromachines (Basel). 2020 May 6;11(5):480. doi: 10.3390/mi11050480 (PMC7281316; doi:10.3390/mi11050480)
Supplement: Supplementary file 1 [file micromachines-11-00480-s001.pdf]

Supplementary Materials: Slicing spheroids in microfluidic device for morphological and immunohistochemical analysis

Satoru Kuriu, Tetsuya Kadonosono, Shinae Kizaki-Kondo and Tadashi Ishida

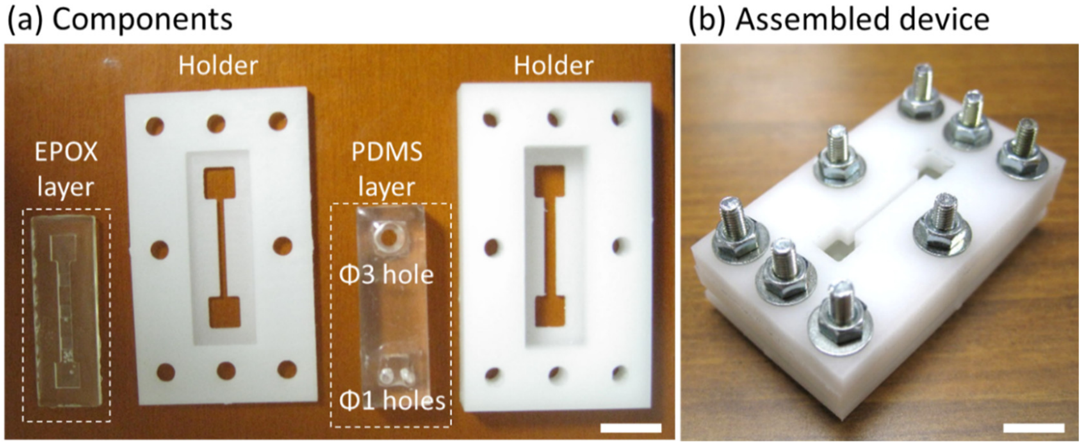

Figure S1. Fabricated EPOX microfluidic device. (**a**) Components of EPOX microfluidic device. (**b**) Assembled EPOX microfluidic device. Scale bar: 10 mm.
